# Heat Shock Proteins in Oxidative Stress and Ischemia/Reperfusion Injury and Benefits from Physical Exercises: A Review to the Current Knowledge

**DOI:** 10.1155/2021/6678457

**Published:** 2021-01-31

**Authors:** Jakub Szyller, Iwona Bil-Lula

**Affiliations:** Division of Clinical Chemistry and Laboratory Hematology, Department of Medical Laboratory Diagnostics, Faculty of Pharmacy, Wroclaw Medical University, Wroclaw, Poland

## Abstract

Heat shock proteins (HSPs) are molecular chaperones produced in response to oxidative stress (OS). These proteins are involved in the folding of newly synthesized proteins and refolding of damaged or misfolded proteins. Recent studies have been focused on the regulatory role of HSPs in OS and ischemia/reperfusion injury (I/R) where reactive oxygen species (ROS) play a major role. ROS perform many functions, including cell signaling. Unfortunately, they are also the cause of pathological processes leading to various diseases. Biological pathways such as p38 MAPK, HSP70 and Akt/GSK-3*β*/eNOS, HSP70, JAK2/STAT3 or PI3K/Akt/HSP70, and HSF1/Nrf2-Keap1 are considered in the relationship between HSP and OS. New pathophysiological mechanisms involving ROS are being discovered and described the protein network of HSP interactions. Understanding of the mechanisms involved, e.g., in I/R, is important to the development of treatment methods. HSPs are multifunctional proteins because they closely interact with the antioxidant and the nitric oxide generation systems, such as HSP70/HSP90/NOS. A deficiency or excess of antioxidants modulates the activation of HSF and subsequent HSP biosynthesis. It is well known that HSPs are involved in the regulation of several redox processes and play an important role in protein-protein interactions. The latest research focuses on determining the role of HSPs in OS, their antioxidant activity, and the possibility of using HSPs in the treatment of I/R consequences. Physical exercises are important in patients with cardiovascular diseases, as they affect the expression of HSPs and the development of OS.

## 1. Introduction

HSPs were accidentally discovered in 1962 by Italian scientist Ferruccio Ritossa who showed an elevated expression of HSP in *Drosophila* [1]. Initially, the manuscript was rejected by a respected journal, which indicated that Ritossa's research was irrelevant to the development of science. Today, we know that it was one of the most important discoveries in the biology. HSPs are the most highly conserved proteins of stress response during evolutionary history and as molecular chaperones are involved in folding of newly synthesized proteins and refolding of damaged or misfolded proteins [2]. HSPs are synthetized in response to different stressors such as heat shock, hypothermia, free radicals, ischemia, hypoxia, ultraviolet radiation, and viral infection [2]. The induction of proteins is remarkably rapid and intense [3, 4].

The major functions of HSPs are assistance in folding of nascent polypeptides [2], prevention of misfolding and aggregation, and protection against apoptotic exchanges [5] as well as participation in suppressing proinflammatory cytokines [6], in intracellular transport [7], and in the modulation of protein expression and cell function [2, 8]. OS is a phenomenon caused by an imbalance between production and accumulation of ROS in cells and tissues and the ability of a biological system to detoxify these reactive products. The concept of OS has been introduced for research in redox biology and medicine in 1985 [9]. OS is of great importance for many life processes and pathophysiology of diseases, e.g., ischemia-reperfusion injury [10]. More and more publications indicate a significant association of HSPs with OS. Molecular and cellular mechanisms are not fully understood. The aim of this review is to present the latest reports on the mechanisms linking the expression of HSPs and the development of OS. We also particularly emphasise its role in I/R injury and the role of exercise on HSP expression recommended especially for patients with cardiovascular diseases.

## 2. Oxidative Stress and Heat Shock Factor: Cellular and Molecular Mechanisms

Increasing ROS levels (and associated development of OS) are counteracted by antioxidant systems including nonenzymatic and enzymatic mechanisms [11]. HSPs are multifunctional proteins that closely interact with the antioxidant [12] and the nitric oxide generation systems [13]. Nitric oxide (NO) is an essential molecule since the excessive formation of NO and generation of peroxynitrite can lead to matrix metalloproteinase-2 (MMP-2) activation, which degrades contractile heart proteins during I/R injury. The administration of MMP-2-inhibitor-NO-donor hybrid normalizes the levels of MMP-2 and peroxynitrite and ameliorates the impact of I/R on the heart [14]. A deficiency or excess of antioxidants modulates the activation of heat shock factor (HSF) and subsequent HSP biosynthesis [15]. The cell protective mechanism called the heat shock response (HSR) maintains protein homeostasis in all eukaryotic cells. HSFs are a family of DNA-binding proteins that regulate gene expression at the level of transcription. The human genome encodes six HSF proteins: HSF1, HSF2, HSF4, HSF5, HSFX (located on the X chromosome), and HSFY (located on the Y chromosome) [16]. HSF1 is the main regulator of expression of protein quality control machinery in response to proteotoxic stress conditions in a multistep activation cycle [17]. HSF1 is constitutively expressed in most tissues and cell types, but it is kept inactive in the absence of stress stimuli [17, 18]. HSF2 is highly expressed during early development of the organism [19]. HSF4 is primarily required in the development of eye lens and is also expressed in the heart, brain, and pancreas [20]. HSF5 function is not exactly known; however, its expression is restricted to spermatocytes [21]. The role of HSFY and HSFX is not fully understood and still poorly characterized. HSFs are highly versatile transcription factors, and HSF1 plays a central role in the HSR as an evolutionarily conserved master transcriptional regulator, which upregulates genes encoding molecular chaperones. HSP expression depends on the transcription factor HSF1, and HSP40, HSP70, and HSP90 prevent this transcription factor from entering the cell nucleus [22]. Recent evidence indicates additional functions of HSF1 and highlights diverse roles such as differentiation, multidrug resistance, and immune response [23]. Oxidative damage of proteins and lipids is also involved in HSP expression, and the OS is considered a key mediator of HSP induction. In eukaryotic cells, HSFs act as transcriptional activators for heat shock genes; HSF1 can be activated by OS and increases the synthesis of protective HSPs [9, 23].

## 3. HSF Activation and Nrf2 Pathway

There is an association between redox homeostasis and HSP expression. A crucial indirect pathway through which ROS activate HSF1 is via oxidation of protein thiols, resulting in the activation of Keap1/Nrf2-Kelch-like ECH-associated protein 1/nuclear factor erythroid 2-related factor 2 and HSP90/HSF-1 transcriptional pathways. Activation of HSF1 or Nrf2 affects the cellular redox state by promoting more reduced environment [24, 25]. Transcription of heat shock genes requires the formation of a homotrimeric HSF1 complex that directly binds to the heat shock promoter element (HSE) present in the promoters of the HSP genes [26]. HSF1 and Nrf2 are critical for adaptation and survival. Both transcription factors participate in the regulation of HSP32 and HSP70 gene expression [27]. HSP70 and HSP90, which are negative regulators of HSF1, inhibit the transcriptional capacity of HSF1 [28]. HSP and HSF1 form a negative feedback loop in which HSP70 expression is induced by HSF1, and HSP70 represses HSF1 activity. Recent research revealed that HSP90 represses HSF1 independently of HSP70 [29], but the mechanism by which HSP90 regulates HSF1 remains unknown. HSP modification may trigger HSF1 release and activation of HSR. Importantly, current reports indicate that the Keap1/Nrf2 pathway is activated first and the HSR second [25]. Klumpen et al. suggested that an early expression of HSP is primarily mediated by ROS and later HSP expression is mainly triggered by protein damage and heat shock factor 1 [22]. Extremely interesting is that HSF1 and Nrf2 may compensate for each other—for example, methionine deprivation was shown to increase the HSP70 expression in an Nrf2-dependent mechanism but independently of HSF1 [30]. This suggests that antioxidant response mediated by the Nrf2 pathway may be a substitute for HSF1-related pathway dysfunction. Lazaro et al. showed that HSP90 inhibition is induced by HSP70 as a result of the activation of its transcriptional regulator and direct association between HSP70 upregulation and Nrf2 activation. It was an important factor related to OS [31]. Nrf2 can also be activated by small HSPs to promote reducing environment.

A direct mechanism indicates that ROS could initiate the formation of disulfide bonds to trigger HSF1 homomultimerization and activation. It is clear that redox regulation of HSF1 multimerization is early and tightly linked stages in HSF1 activation, but the process is dramatically inhibited under conditions of hypoxia or in the presence of reducing agents [32]. The direct activation of HSF1 by H_2_O_2_*in vivo* strongly suggests that HSF1 directly senses these stresses via changes in redox state, and activation of Nrf2 involving Cys35 and Cys105 strongly suggests that these two cysteine residues are engaged in disulfide bonds, the formation of which is essential for HSF1 homomultimerization [32]. This supports a mechanistic relationship between changes in cellular redox state and conditions that cause activation of the HSR. Nrf2 can also be activated via HSF1-mediated induction of p62, a classical receptor of autophagy, because p62 can displace Nrf2 from Keap1 [33]. From a different point of view, activating mutations in Nrf2-Keap1 are frequently found in human cancers which are associated with aggressive growth and resistance to therapies. Baird et al. showed that Nrf2 target genes metabolize the quinone-containing geldanamycin compounds into more potent HSP90 inhibitors. They can enhance their cytotoxicity while simultaneously restricting the lethal effect on cells with aberrant Nrf2 activity, resulting in cell death [34]. This is a different approach to understanding the interaction network between HSP and OS. An association between HSF1, Nrf2, and the redox state is very strong. In the absence of HSF1, expression of several HSPs is downregulated, simultaneously with an increased level of OS and ROS-induced oxidative damage [9]. HSF1 can also induce sets of genes to protect against the OS caused by peroxides, but the exact mechanism is unknown [35]. Thus, HSF1 is an important element of the antioxidant system. By increasing detoxification pathways and antioxidant potential, Nrf2 is involved in the protection of cardiac fibroblasts and cardiomyocytes against OS [36]. The cardioprotective function of Nrf2 in I/R injury results from an activation of the prosurvival PI3K/Akt kinase (phosphoinositide 3-kinase/protein kinase B) pathway which was shown to play a role in mechanisms of increased myocardial tolerance [37]. Wang et al. have also reported that the regulation of redox imbalance induced by I/R injury was associated with modulation of Nrf2 [38].

## 4. HSF Activation through Hypoxia-Induced Factor

Another mechanism of HSF activation involves hypoxia-induced factor (HIF). HIF-1*α* is one of the critical regulators of HSF1 and is essential for the activation of HSR [39]. Hypoxia response elements for the HIF-1*α* and HIF1*β* in the promoter region of the HSF1 gene are observed. This strongly suggests a regulatory role for HIF-1*α* in HSF1-mediated HSR [39] and may be especially important in the aspect of I/R. HSF1 level showed a reduction in cells transiently silenced for HIF-1*α*, suggesting a significant role for HIF-1*α* in the expression of HSF [39].

## 5. Heat Shock Proteins and Oxidative Stress

The function of HSP in maintaining the oxidative-antioxidant balance seems to be multidirectional. Nowadays, the effect of HSP on OS has been considered in the aspect of cellular homeostasis [40], atherosclerosis/oxLDL (oxidized low-density lipoproteins) [41], pollution [42], hearing loss [43], and many others. Interestingly, HSPs have been reported to work hand in hand with the antioxidant system to inhibit or neutralize the cellular effects of ROS [44]. For example, the best known HSP70 family promotes an increase in free 20S proteasome and therefore increases the capability to degrade oxidized proteins. It prevents the accumulation of oxidized proteins and directly promotes their degradation by the 20S proteasome [12]. HSP70 plays an important role in the pathophysiology of diseases related to air pollution and OS. Baldissera et al. showed that exposure to air pollution causes significant increase in plasma HSP70, marked by 29% higher levels of the HSP72 form. An increase in the extracellular-to-intracellular HSP70 ratio (H-index) was related to elevated activity of superoxide dismutase (SOD) and increased content of the carbonyl group [45]. Data also showed a redox imbalance in the plasma that occurs concomitantly with increased levels of extracellular HSP70 (eHSP70). Another important link between HSP and OS is that the *HSP70-2* polymorphism is related to ROS levels and appears to have a role in the different expressions of HSP70-2 (a sensor for the redox status of the cells) under oxidative stimulus [46]. HSP and OS interaction can even be related to a pineal hormone—melatonin—which is considered a potent candidate in the regulation of oxidative damage [47]. After melatonin treatment of H_2_O_2_-stressed fish hepatocytes, a significant decrease in SOD and catalase (CAT) activity, glutathione (GSH), and malondialdehyde (MDA) level, as well as in HSP70 and HSP90 level, was observed [48]. The level of Akt and ERK1/2 (extracellular signal-regulated kinase 1/2) in hepatocytes was increased, and a positive correlation with H_2_O_2_ concentrations was reported [48]. This indicated the protective efficacy of melatonin against OS. HSP can regulate ERK1/2 in the MEK-ERK (mitogen-activated protein kinase) pathway [49] and can also interact with Akt [50]. Formation of the Akt-HSP complex stabilizes Akt that protects the cells from apoptosis [51]. There is a strong relationship between OS and activation of the ERK1/2 or Akt pathway [52]. Very interesting work of Klumpen et al. concluded that elevated ROS formation and/or reduced GSH buffer capacity (which caused higher fluctuation frequencies of ROS) accelerate the expression of HSPs for earlier cell component protection [22]. It is known that HSPs may be involved in regulating the function of SOD1. Mutations in HSP27 may be related to the inability to prevent SOD1 aggregation [53]. Similar relationship may exist for the HSP70/HSP40/HSP110 machinery in aggregate disassembly in reference to SOD1 [54]. Xia et al. showed valuable data from gasoline filling station workers [55]. Workers exposed to benzene, toluene, ethylbenzene, xylene, and manganese presented decreased activity of SOD and glutathione peroxidase (GPx) but significant increase in the level of MDA compared with the control group [55]. Authors indicated that lower levels of SOD and GPx might be an early warning signal of oxidative damage. At the same time, plasma HSP70 was significantly higher in the exposed group than in the control. A positive correlation between MDA and HSP70 concentration and a negative correlation between HSP70 and SOD/GPx activity were observed [55]. The authors concluded that OS may damage the normal antioxidant enzyme system and induce an increase in HSP70 to prevent cells. On the other hand, HSP70 regulates the cellular redox status. Blocking the JAK2/STAT3 (Janus kinase 2/signal transducer and activator of transcription 3) signaling pathway promotes OS and cell apoptosis via the downregulation of HSP70 [56]. HSP27 has many additional functions. Overexpression of HSP27 and *α*B-crystallin induces a dose-dependent increase in glutathione levels. Small HSPs have been also correlated with an increased level of iron, a catalyzer in the Fenton reaction [57]. Other small HSP—HSP20 (HSPB6)—shows multifunctional protective roles in multiple organs [58]. The protective role of HSP20 as an antioxidant agent [59] was illustrated among others in cardiovascular diseases and I/R injury [60]. HSP22 was induced by OS and was drastically elevated in airway epithelial cells (AECs) after ozone exposures, thus protecting AECs from oxidative injuries through the Nrf2-NQO-1 (NADPH quinone acceptor oxidoreductase 1) [61]. Similarly, OS-induced expression of HSP20 in AECs enhanced the translocation of Nrf2 and subsequently increased the expression of NQO-1 [61]. HSP90 plays important roles in cell survival as an inhibitor of programmed cell death during OS [62]. Since iron plays a role in the development of OS in the Fenton reaction, HSP90 has been identified as iron-binding protein in the HeLa cell membrane [63]. HSPs have been also shown to interact with lipids. The latest research indicates that the HSP90 is able to bind to oxidized phospholipid and prevent their further oxidation to secondary products, functioning as a scavenger of oxidation products. Zhang et al. showed that (i) HSP90 prevents phospholipids and oxidized phospholipids from further oxidation to more pathogenic and reactive end products [64], (ii) HSP90 can scavenge the DPPH-(1,1-diphenyl-2-picrylhydrazyl) and ABTS-radical (2,2′-azino-bis-3-ethylbenzthiazoline-6-sulphonic acid), wherein the scavenging ability of HSP90 for DPPH is higher than that of the glutathione [65], and (iii) HSP90 was effective in the scavenging of hydroxyl radical—a highly reactive molecule, one of the major causes of OS [65]. HSP90 and HSP70 can also bind with oxidation products of arachidonic acid (polyunsaturated fatty acid present in the phospholipids of cell membranes) which play a role in the development of several diseases [66, 67]. OS alters HSP90 expression in endothelial cells, inducing its surface localization, and promotes the upregulation of HSP90 surface expression on cells, thus rendering the protein a possible target of autoimmune reactions [68]. Therefore, HSPs play a wide role, positively or negatively depending on their involvement in various cellular pathways.

## 6. Heat Shock Proteins in Ischemia/Reperfusion Injury

**Figure 1:**
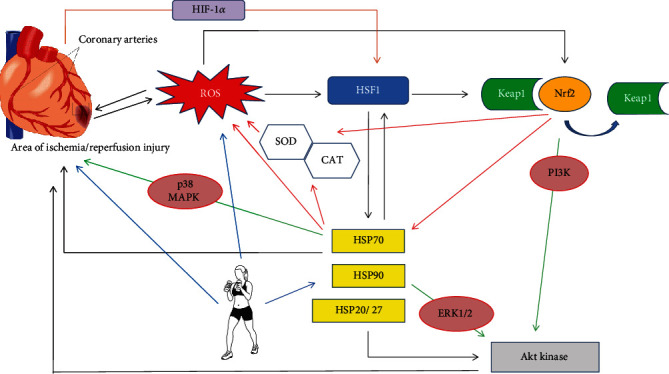
The role of HSPs in oxidative stress and I/R injury—selected signal pathways. Ischemia/reperfusion (I/R) leads to the production of large amounts of reactive oxygen species (ROS), which can lead to further cellular damage. Both ROS and hypoxia (through hypoxia-induced factor) can activate the transcription factor HSF1 (heat shock factor 1). Activation of HSP gene expression involves the stress-inducible conversion of HSF1 from the inactive monomer to the DNA-binding competent homotrimer. HSF1 activates Nrf2 (nuclear factor erythroid-derived 2-like 2) through increased expression of p62 (p62 not shown). The cardioprotective function of Nrf2 in I/R injury results from an activation of the prosurvival PI3K/Akt (phosphoinositide 3-kinase/protein kinase B) kinase pathway but also from activating antioxidant systems. New data revealed a potential crosstalk between Keap1/Nrf2 (Kelch-like ECH-associated protein 1) and Hsp90/HSF1 cytoprotective pathways and the possibility of their comodulation. Other cellular pathways involved in ROS-HSPs-I/R interactions are ERK1/2 (extracellular signal-regulated kinase 1/2) and p38 MAPK (p38 mitogen-activated protein kinases). In addition, HSPs may interact with antioxidant systems or function “hand in hand”. Physical exercises increase both the expression of HSPs and, on the one hand, the formation of ROS and affect the oxidative-antioxidant balance. SOD: superoxide dismutase; CAT: catalase.

ROS are key players in normal cardiovascular physiology and cell signaling, but the OS plays an important role in the development of cardiovascular diseases [69]. The possible role of HSPs in the protective mechanisms of I/R injury has been postulated for a long time. Cardiac pathologies induce changes such as cellular redox status or calcium homeostasis impairment, which in turn can induce misfolding of proteins. This can lead to the formation of proteotoxic soluble peptides [70]. The cardioprotective effect of HSPs is manifested in an increased cell resistance to hypoxia [71] and oxidative stress [72] and in increase in functional recovery with a decreased infarction size after experimental induction of I/R [73, 74]. The restoration of blood flow after ischemia leads to massive production of ROS, which generate severe damage to biomolecules—a phenomenon called myocardial reperfusion injury [10]. Most studies concern two main families of HSPs: HSP70 and HSP90. Animal models demonstrated an increased expression of HSF1, resulting in an increase in HSP70 and HSP90 mRNA levels, wherein the maximum level was detected during reperfusion and the increase in HSP70 was much higher than that in HSP90 [74]. HSP90 has recently been investigated as a novel target to reduce I/R injury. A very interesting work concerns the use of the HSP90 inhibitor (HSP90i) in cardioprotection in heart transplantation. Heart pretreatment with HSP90i during cardioplegia reduced infarct size, fibrosis, and macrophage infiltration in a nonreperfused cardiac ischemia model [75]. In a circulatory death model of donation, HSP90i protected against functional loss, reduced infarct size and cell damage following warm ischemia, reduced cellular stress (as indicated by the Bax/BCL-2 ratio), and induced the expression of key antioxidant enzymes such as SOD1 and CAT [76]. At this point, it should be mentioned that CAT is a major enzyme involved in the detoxification of hydrogen peroxide. It has been reported that in rats, exposure to heat shock leads to an increase in myocardial CAT activity and this observation correlated with an improvement in function of the rat heart after low- and no-flow ischemia [77]. One of the first studies (1988) indicated that hyperthermic treatment may be therapeutic for salvaging an ischemic myocardium during reperfusion, through a mechanism involving increased levels of myocardial catalase [77]. Similar relationships were demonstrated a few years later by Wall et al. [78]. Current research confirmed previous observation and was carried out, e.g., on a liver and lung model where direct delivery of polyethylene glycol-catalase (PEG-CAT) during normothermic *ex vivo* lung perfusion significantly reduced I/R injury [79] and intrahepatic delivery of PEG-CAT during I/R significantly reduced the activity of alanine aminotransferase and aspartate aminotransferase and concentration of MDA and GSH [80]. Interestingly, the activity of CAT in the heart is very low, and this may be a factor responsible for high sensitivity of this organ to I/R injury [81]. In turn, CAT, as an antioxidant enzyme, can protect against I/R injury. It seems that there are no reports describing a clear relationship between HSPs and CAT activity in humans in terms of I/R injury. An increase in HSP expression and CAT and SOD activity in response to ischemia in a partial hepatectomy rat model [82] or in *Drosophila melanogaster* in response to stress factors has been observed [83]. It is difficult to refer to studies describing a relationship between two parameters and the direct molecular mechanism in I/R injury in humans. HSP90 inhibitors activate Nrf2 transcription and induce antioxidant response element activity that reduces cellular OS [76]. As indicated by the authors of the study, it is also necessary to investigate the electrophysiological effects. HSP70 also shows a strong cardioprotective effect, and it can become a promising therapeutic target [84]. Song et al. indicated that rat cardiomyocytes subjected to oxygen-glucose deprivation/reperfusion showed increased expression of HSP70 and p-p38 MAPK; the same observation applies to increased HSP70 expression and phosphorylated p38 MAPK during I/R-induced myocardial injury, and inhibition of HSP70 by quercetin significantly increased myocardial infarct size [85]. It is very important that HSP70 inhibition led to upregulation of p-p38 MAPK and p-STAT3 and downregulation of SERCA2 (sarco/endoplasmic reticulum Ca^2+^-ATPase) during myocardial I/R injury, and inhibition of p38 MAPK phosphorylation attenuated effects induced by HSP70 inhibition [85]. In contrast to inhibition of HSP90, inhibition of HSP70 aggravates [Ca^2+^]*_i_* overload, apoptosis, and inflammation through regulating p38 MAPK signaling during I/R cardiac injury. Other studies point to the involvement of other pathways, e.g., HSP70 and Akt/GSK-3*β* (glycogen synthase kinase 3)/eNOS (endothelial nitric oxide synthase) [86], protection by regulating HSP70 expression via activation of the JAK2/STAT3 pathway [87], or activation of the PI3K/Akt/HSP70 signaling axis [88]. This data indicates that the HSP interaction network is extremely complex (Figure 1). Both HSP70 and HSP90 directly interact with hERG (the human ether-á-go-go-related gene) whose disorders can lead to long-QT syndrome and sudden cardiac death [89]. Disturbances in the stability of the cell membrane can occur during myocardial ischemia in the course of myocardial infarction. HSPs modulate mitochondrial function as well as ROS generation during OS. As now known, OS may also play a significant role in the occurrence of cardiac arrhythmias affecting biomolecules and ion channels, leading, e.g., to atrial fibrillation [90]. HSP22 plays an important role in the adaptation of the myocardium in response to OS. It was found to be very increased in the cardiac OS conditions, and the overexpression of this protein protects the heart against ischemic damage by inducing the expression of inducible nitric oxide synthase (iNOS) [91]. This is an extremely important observation, as indicated by the authors of the latest work of Wu et al. The authors pointed that cardioprotection by NO donor drugs (like nitroglycerin) has been limited and stimulating the formation of NO through endogenous iNOS might have a better biological effect than that provided by NO donors [92]. Beyond ROS inhibition, HSP27 interacts with Akt maintaining the kinase in an active conformation state and decreases the mRNA levels of tumor necrosis factor-*α* and interleukin-1*β* [93, 94]. Increased HSP20 expression protected against I/R injury in animal studies of an isolated rat heart at a Langendorff apparatus, resulting in full functional recovery, reduced infarction, and protection against myocardial apoptosis by regulating the B-cell lymphoma-2 (Bcl2)/Bcl2-associated X (Bax) ratio and inhibiting caspase-3 activation. These implicate this HSP20 as a potential therapeutic target for ischemic heart disease [60, 95]. HSPs can also form a complicated network of pathways that contributes to the reduction of ROS accumulation and improvement of calcium homeostasis [84]. The mechanisms of HSP70 action are related to inhibition of apoptosis and OS [84]. Myocardial HSP70 activates mitochondrial Mn-SOD, which is associated with mitochondrial protection and reduction of apoptosis [96]. In addition, HSP70 is expressed in the brain in the early stages of ischemic injury which indicates an important protective mechanism [97]. It has been also suggested that HSP70 decreases ROS level in cardiomyocytes resulting in an inhibition of the activation of the downstream TAK1/AMPK (transforming growth factor-*β*-activated kinase 1/AMP-activated protein kinase) cell death pathway [98]. It is very important that HSPs are released into the extracellular environment or enter the systemic circulation under stress conditions. Extracellular HSPs can act as a form of communication during injury and as immunological regulators that potentiate the innate immune response [99]. Extracellular HSPs may have the potential as diagnostic biomarkers of myocardial infarction. Modulation of the protein-protein interactions between HSP complexes is a promising therapeutic strategy against myocardial infarction (MI). During MI, HSPs participate in the protection of cardiac troponin I and T from degradation, stabilization of the mitochondrial membrane, and protection of cardiomyocytes from I/R injury [94]. It seems that HSPs play an important role in the treatment of myocardial ischemia, and their action is multidirectional and complicated. HSPs are also crucial for renal cell response to ischemic injury, and HSP70 is a highly sensitive marker of I/R injury [100]. There are some interesting reports on the use of hyperbaric oxygen therapy (HBOT) in the treatment of ischemia, describing the role of HSP expression and reduction of ROS biosynthesis during reperfusion. HBOT promotes protection by upregulating HSPs via an increase in Nrf2-mediated antioxidant gene expression and upregulates essential proteins involved in intracellular GSH production and transport [101, 102]. The findings have provided new evidence to support that HBOT induces tolerance to I/R injury by upregulating HSP activity. HSPs are also involved in the proper function of the endothelium and the development of inflammatory response. It is extremely important to know the details of these interactions and use them, e.g., in the treatment of COVID-19 [103, 104]. It is also well demonstrated that I/R injury is associated with increased activity of MMP-2 during OS. Therefore, modulation of HSP expression (as above), together with interference in the expression of iNOS and eNOS during OS, may provide benefits in the prevention of I/R injury [105].

It has been confirmed that plasma exosomes contain HSP70, indicating its role in the modulation of ROS [106]. HSPs play a vital role in ameliorating ROS-dependent damage in neurodegenerative disorders [107, 108] and I/R injury. Exosome-mediated specific delivery of HSP70 attenuated I/R injury by reducing generation of ROS through the increase in SOD activity, enhanced mitochondrial membrane potential, and restored mitochondrial function, inducing overexpression of HSP70 in the ischemic region and maintaining the integrity of mitochondria [109]. Recently, much attention has been paid to the ratio of iHSP to eHSP (intracellular-to-extracellular HSP). Given the anti-inflammatory nature of iHSP70 and inflammatory nature of eHSP70, it is assumed that the ratio *R* = [eHSP70]/[iHSP70] = 1 for the controls of a given condition and values higher than 1 indicate a greater proinflammatory response [110]. In diabetes mellitus, eHSP72 levels appear to be elevated due to adiposity and inflammation, whereas iHSP72 levels appear to be decreased [111] while intensifying OS [112]. The role of ROS in the development of the inflammatory process is well established. ROS activates nuclear factor kappa B (NF-*κ*B) via extracellular signal-regulated kinases, c-Jun N-terminal kinases (JNKs), p38 MAPK, PI3K/Akt, and others [113, 114], which in turn lead to increased expression of matrix metalloproteinase-9, cyclooxygenase 2, IL-1, and IL-8. HSP70 and HSP32 (heme oxygenase 1) can protect cells and tissues from the deleterious effects of inflammation [115].

## 7. Heat Shock Proteins, Oxidative Stress, and Physical Exercise

There are numerous reports, including meta-analysis, on the influence of exercise on the oxidative-antioxidant balance and the expression of HSPs. They indicated that exercise training reduces cardiovascular mortality [116–118] and has been frequently attributed to the reduction of classical cardiovascular risk factors including OS [119, 120]. The effect of exercise on HSPs depends on age [121], sex [122], and HSP subtype [123]. Physical exercise exerted potent impacts on the myocardial antioxidant defense system and decreased cardiac damage [124]. Aerobic exercise is associated with a cardioprotective phenotype, but the exact mechanisms responsible for this phenomenon remain unclear. Exercises induce increase in antioxidant capacity of cardiomyocytes through upregulation of SOD and catalase, as well as overexpression of HSP70 [125]. Endurance exercise training elevates myocardial HSP72 by even 400-500% in young adult animals and is associated with a reduction in I/R injury in the heart [126]. Chronically elevated basal levels of HSP70 were found in cardiac tissue of trained mice, and what is interesting, an acute treadmill running did not induce a further increase [127]. Animals that have been submitted to 40 minutes of physical activity showed increased expression of HSP70 in the heart [128]. L-Arginine (biological precursor of NO) and a treadmill exercise program exerted more potent increase in the expression of HSP70; an increase in the total antioxidant capacity (TAC) and SOD and CAT content in the L-arginine and exercise group was also observed [129]. Elevated cellular HSP72 can protect the myocardium against I/R injury by repairing unfolded proteins (chaperoning activity) and by stabilizing the function of the endoplasmic reticulum via HSP70-related autophagy [130]. Exercise-induced increase in Mn-SOD activity attenuated I/R-induced oxidative modification of Ca^2+^-handling proteins and resulted in decreased cardiomyocyte death [131]. Ahn showed that after a 12-week exercise program, HSP70 and SOD1 expressions in the myocardium were significantly higher in the exercise group compared to the control group [132]. Wang et al. showed that HSP70 expression level in the brain of rats from the 5-week long aerobic exercise group was 52% higher than that in the control group [133]. Hydroxyl radical scavenging capacity, SOD, and GPx were also significantly higher than those in the control group [133]. The authors concluded that HSP70, similar to other powerful antioxidant and repair proteins, can intervene in the oxidative damage caused by oxidative radicals. Another study indicated that exercise training for 14 weeks reduced MDA and carbonyl protein concentrations but HSP70 and TAC were increased significantly after exercise training [134]. During acute exercise, various HSPs are upregulated in organs and tissues. Apart from activating the HSF1, exercise can induce activation of the adrenergic receptor-mediated signaling kinase which inhibits the ERK1/2 pathway and leads to the increase in HSP70 concentrations [135]. Apart from interacting with the antioxidant system, HSPs exert their direct anti-inflammatory effect through interaction with NF-*κ*B and blocking its activation, which may be important in I/R injury [136]. Elevation in iHSP70 may inhibit JNK-dependent signal transduction therefore promoting cell survival [137]. The balance between iHSP70 and eHSP70 (i.e., iHSP70/eHSP70 ratio) will modulate NF-*κ*B translocation capacity and then the inflammatory level [110]. Exercise training did however induce the interaction between HSP90, 5′AMP-activated protein kinase (AMPK), and eNOS in the hearts, and this network is complicated; HSP90 is a regulator of eNOS activity and promoted eNOS coupling while AMPK influenced the coupling of eNOS by promoting its interaction with HSP90 [138, 139]. Considering the above, it can be concluded that physical exercise plays an important role in the prevention of cardiovascular diseases by induction of HSP expression and modulation of OS, including the NOS/NO-related system.

## 8. Conclusion

Heat shock proteins play a cytoprotective role under pathological conditions such as cardiovascular diseases. The knowledge about cellular and molecular mechanisms underlying ROS-mediated modulation of HSP expression can help to better understand the pathophysiology of OS, which is associated with the development of many diseases (cardiovascular, neurodegenerative, etc.). I/R injury is considered a major contributor to tissue damage in multiple clinical situations such as myocardial infarction, stroke, and organ transplantation. Oxidative damage is a key factor in the initiation of I/R. HSP expression is highly sensitive to I/R injury. Understanding the exact mechanisms of HSP and the structure of the protein interaction network can help to better understand the pathophysiology and treatment of many diseases, as well as to develop new drugs. There is a need to understand the relationship between cell pathways—signaling, metabolism, etc. The relationships between HSP and OS discussed in this work seem to be very complicated and not yet fully understood. Data showed that modulation of HSP expression in reperfusion injuries may result in better treatment of myocardial infarction. This can also help to prepare organs for the transplantation.
